# Supplementary material for: Conditional chemoconnectomics (cCCTomics) as a strategy for efficient and conditional targeting of chemical transmission
Source: eLife. 2024 Apr 30;12:RP91927. doi: 10.7554/eLife.91927 (PMC11060718; doi:10.7554/eLife.91927)
Supplement: Figure 3—source data 3. [file elife-91927-fig3-data3.zip › Figure 3 source data3 part 2.zip!/Figure 3-Source data 3-part2/1060f2-1-CRISPResso_Report_gl5umwyq.zip!/CRISPResso_on_1060f2_2/3b.Insertion_deletion_substitutions_size_hist.pdf]

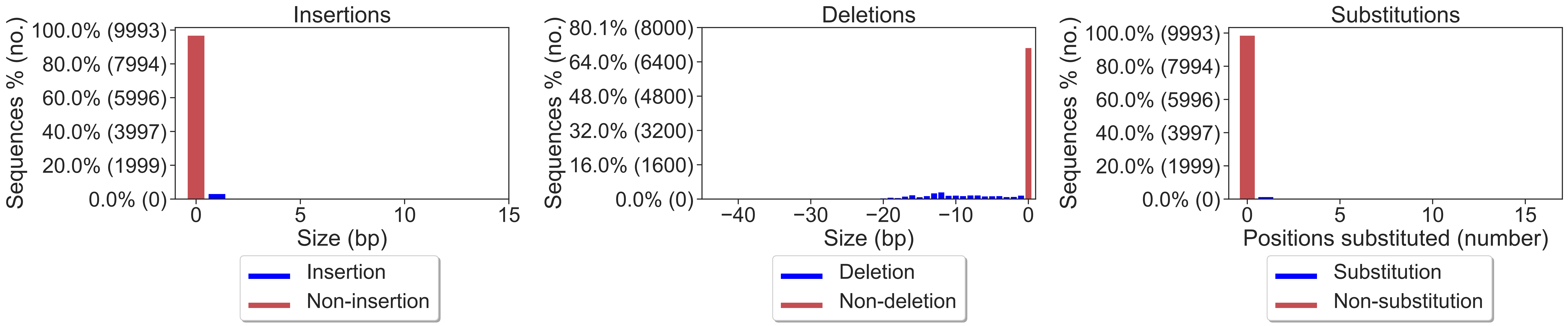

Insertions
Sequences % (no.)
100.0% (9993)
80.0% (7994)
60.0% (5996)
40.0% (3997)
20.0% (1999)
0.0% (0)
0
5
10
15
Size (bp)
Insertion
Non-insertion
Deletions
80.1% (8000)
64.0% (6400)
48.0% (4800)
32.0% (3200)
16.0% (1600)
0.0% (0)
−40
−30
−20
−10
0
Deletion
Non-deletion
Substitutions
Positions substituted (number)
Substitution
Non-substitution
